# The Teaching Brain: Beyond the Science of Teaching and Educational Neuroscience

**DOI:** 10.3389/fpsyg.2022.823832

**Published:** 2022-03-08

**Authors:** Giancarlo Gola, Laura Angioletti, Federico Cassioli, Michela Balconi

**Affiliations:** ^1^Department of Education and Learning, University of Applied Sciences and Arts of Southern Switzerland, Locarno, Switzerland; ^2^International Research Center for Cognitive Applied Neuroscience (IrcCAN), Università Cattolica del Sacro Cuore, Milan, Italy; ^3^Research Unit in Affective and Social Neuroscience, Department of Psychology, Università Cattolica del Sacro Cuore, Milan, Italy

**Keywords:** neuroeducation, Teaching Brain, teacher education, teacher development, two-persons neuroscience, hyperscanning, brain-to-brain coupling

## Introducing the Teaching Brain

The field of neurodidactics studies has amplified knowledge about learning processes, focusing on the learning subject and the implications for teaching-learning brain (Goswami, 2004; Strauss, 2005; Battro, 2007, 2010; Fischer, 2009; Fischer and Daniel, 2009; Geake, 2009; Immordino-Yang, 2016; Willingham, 2017; Tibke, 2019).

The data and research supported by neuroscience that invest at different levels the theories and practices of education are not new, by way of example: Iran-Nejad et al. (1992) stressed the value of a biological perspective in education; Jensen (2005) connects the role of different cognitive processes (such as emotions, attention, motivation, and rewards) to learning processes (e.g., he discusses why the stress response impedes learning), Willingham and Llyod (2007) describe four techniques to integrate neuroscientific data into the psychological theory of educational constructs (such as reading), and Schwartz (2015) presented the evolution of research at the intersection between neuroscience and education.

The rapidity with which attention to educational neuroscience and the relationship between education and the brain has evolved, led to the development of neuromyths (Geake, 2009; Sousa, 2011). Neuromyths have been defined as “misconceptions generated by a misunderstanding, a misreading or a misquoting of facts scientifically established (by brain research) to make a case for use of brain research in education and other contexts” (OECD, 2002, p. 69). To mention a few, they include the polarization of brain hemisphere specialization and its relationship to learning, the misconception that brain plasticity is relative only to specific critical events, the idea that learning improves or is facilitated under conditions of higher synapses (Goswami, 2004; Santoianni, 2019), the use of only “10% of our brain”, the acquisition of information about specific preferred learning styles (Howard-Jones, 2014). To establish a correct dialogue between neuroscience and education, these misconceptions should be explored, as done by recent studies (e.g., Hermida et al., 2016), and addressed in practice with the appropriate information, neuroscience courses, or training.

Furthermore, the idea that neuroscience can inform and potentially influence education raises controversy and open debate (Ansari et al., 2012). Meirieu (2018) argues that the neuroscientific approach would only be able to visualize the existence of a human being through brain activity and the image of the mind, but not the content that forms and sustains thought and generates knowledge. One of the major criticisms often concerns the artificiality of neuroscientific experiments that cannot be easily applied to real educational contexts, neuroscientific knowledge is far removed from classroom interactions, some facets of neuroscience are not relevant to some facets of classroom learning (Bruer, 1997; Colvin, 2016).

To overcome this limitation, cognitive applied neuroscience research started to apply neuroscientific tools in highly ecological contexts, such as the classrooms (Brockington et al., 2018). Also, in the two-person (teacher-student) educational neuroscience, the relatively newborn hyperscanning paradigm in neuroscience, that involves capturing the brain activity of two or more participants engaged in interactive activities at the same time (Babiloni and Astolfi, 2014; Balconi and Vanutelli, 2017; Crivelli and Balconi, 2017) may allow researchers to grasp the relation between the teacher and the learner (or even the class group) (Dikker et al., 2017; Pan et al., 2020). Hyperscanning can be defined as a method that allows for the performance of human behavioral experiments in which participants can interact with each other while brain neuroimaging data is acquired in synchrony with the behavioral interactions (Montague et al., 2002).

Accordingly, although the provocation of the French philosopher (i.e., Meirieu), still feeds widespread neuroscepticism (see for example the Editorial, 2005; Bowers, 2016; Krammer et al., 2021), it finds some opposite positions and evidence in several recent studies that support the blending of neuroscience and education as an engine of knowledge (Thomas, 2019; Davidesco, 2020; Davidesco et al., 2021). According to this view, the knowledge of how the brain works and its anatomy could contribute to (i) the understanding of teaching-learning processes, and (ii) the identification of learning environments oriented to promote and support neuroplasticity and that could be facilitators of the learning process. Several studies link brain plasticity with the ability to learn, explaining how this is closely dependent on changes related to the architecture and chemistry of our brains (Caine and Caine, 2006).

Despite this multiplicity and the prevailing focus on the brain of the learner and learning as a cognitive function and neural processes, relatively few studies to date have specifically addressed a deeper understanding of the teacher's perspective according to the neuroscientific approach of the Teaching Brain (TB) (Fischer and Rose, 1998; Battro, 2010). Some works have been conducted specifically in the field of cognitive neuroscience and experimental psychology on the topic of TB exploring the basis of cognitive processes supporting teaching practices (Pasquinelli et al., 2015; Calero et al., 2018; Corriveau et al., 2018), compared to imaging and traditional neuroscience that focuses on a traditional localization approach. However, we believe that a two-person neuroscientific approach such as that of hyperscanning can best grasp the nature of TB, converging information on brain localization, cognitive and emotional processes, and student-teacher interactional dynamics. TB is a concept that reflects the complex, dynamic, and context-dependent nature of the learning brain, in fact, teaching is an interaction between two entities: the teacher and the learner (Rodriguez, 2013).

The next section will present the neuroscientific research on the topic of the teacher's brain and its interconnections with the teaching-learning relationship and teaching practices. Finally, we discuss how novel paradigms of neuroscience might account for and deepen the role of TB in the teaching-learning process, which is conceived in an interactive dynamic.

## Neuroscientific Studies on the TB

### Empirical Studies Analyzed on the TB

Without claiming to be exhaustive, the following paragraphs will describe the studies that, in line with the new trends in cognitive neuroscience, aimed to apply the neuroscientific tools outside the laboratory, directly in the classroom, and which used the hyperscanning paradigm to grasp the interactive dynamic between teacher and student. Unlike the two recent essays by Davidesco (2020); Davidesco et al. (2021), this contribution focuses on hyperscanning studies involving the figure of the teacher. The findings reported below might be particularly interesting for a better understanding of the TB perspective.

Research on the TB includes, in particular, those by Rodriguez (2013); Rodriguez and Solis (2013), and highlights how the teacher's brain is able to process student-centered information, forming a theory of student cognition that considers what the students are thinking and the knowledge they would be able to acquire and accumulate. Authors suggest teachers can, therefore, use this model to guide not only what the students are thinking and learning, but also what they would be capable of (Rodriguez, 2013). For instance, authors suggested that developing a theory of the student's mind, cognition, emotion, and memory allows teachers to tailor their demands to the student and invite them to learn what they deem impossible to learn. However, it must be clarified that the ability of the teacher to adjust its requests based on a representation of the student's learning brain has not been measured or verified experimentally.

In the two-person educational neuroscience framework, neuroscientific research by Holper et al. (2013) and Dikker et al. (2017) attempt to identify the cortical correlates involved in teacher-student interactions during the performance of a teaching model. The evidence reported by brain studies solicit relevant pedagogical variables in student-teacher interaction. Using the technique of functional Near-Infrared Spectroscopy (fNIRS), the research team of Holper et al. (2013) examined the hemodynamic brain correlates of teachers and students during a specific teaching activity based on Socratic dialogue and, concerning the TB, found that higher teacher-student brain synchrony positively correlates with dialogs in which the student transferred the learned knowledge. In a comparable way, but using the portable electroencephalogram (EEG) technique, Dikker et al. (2017) study was conducted attempting to neuroscientifically detect the synchronic relationship between teachers and students by recording, repeatedly over several days during a semester, the brain activity of a group of students and the teacher simultaneously while they were in the classroom. The results suggested that brain-to-brain synchrony is a sensitive marker that can predict both classroom engagement (students rated as more engaging watching videos and group discussions over listening to the teacher reading aloud or lecturing) and classroom social dynamics (in terms of social closeness ratings and social interactions) and that this relationship may be driven by shared attention within the group.

Also, Bevilacqua et al. (2019) adopted an EEG-hyperscanning paradigm and showed that social factors (e.g., perceived closeness) are reflected in the brain-to-brain synchrony of a student-teacher dyad and can predict cognitive outcomes such as students' academic performance. While Bevilacqua et al. (2019) found no link between student-to-teacher brain synchronization and students' performance on a knowledge test, two other research found the opposite (Cohen et al., 2018; Davidesco et al., 2019). For example, Davidesco et al. (2019) found that neural synchronization between students and between students and teachers predicted students' performance on a test given a week following a lecture.

To narrow the gap between experimental research conducted in the laboratory and the classroom environment, Brockington et al. (2018) initiated a series of fNIRS hyperscanning studies directly in the classroom to capture the brain activity and derived physiological phenomena of students and teachers in the typical realistic scenario of the educational relationship (i.e., in the first experiment the dyad was performing an interactive board game; in the second and third experiment, the students were attending a lecture). In their first study, authors revealed a close and positive correlation between a teacher's and a student's activation pattern [oxygenated hemoglobin (HbO) concentration changes] in the prefrontal cortex (PFC) during an educational interaction, which can be associated with a phase of monitoring of the student's actions by the teacher, which is crucial to verify the completion of an educational task. More interestingly, a positive correlation between the teacher's temporoparietal junction (TPJ) and the student's PFC changes in HbO was detected, demonstrating that the learning process is always a bidirectional transfer of knowledge, in which the student activates brain regions involved in high order cognition (e.g., PFC) and the teacher recruits parietal areas (e.g., TPJ) supporting social processes, like the capability of empathizing and perspective-taking. With the appropriate cautions, the neuroscientific correlations between the regions supporting mental processing in the teacher and higher cognition processes in the students highlighted by the group of Brazilian researchers (Brockington et al., 2018) suggested the neural-based bidirectional transfer of knowledge featuring this interaction and draw a synergy with the dynamic interaction models of the mind of the teacher and the learner (see Rodriguez and Fitzpatrick, 2014).

More recently, Pan et al. (2020) employed fNIRS hyperscanning to assess simultaneously the neural responses deriving from teacher-learner couples during two dynamic conceptual learning approaches (scaffolding vs. explanation approach). In this study, the scaffolding behaviors included asking guiding questions or providing hints, while the explanation approach comprised providing definitions or clarifications. The findings indicated that brain-to-brain coupling was linked with learning outcomes, and, more importantly, was driven by the teacher's scaffolding behaviors and not from an explanation approach. This evidence shows that, as a pedagogically relevant metric, the brain-to-brain coupling is associated with the naturalistic educational process during instructor-learner contact when engaged in a constructive dialog, but not when the teacher is providing clarifications or information.

Although taking place in situations as real as possible of teaching-learning, the research conducted by Dikker et al. (2017); Brockington et al. (2018), and that of Pan et al. (2020) have adopted very rigid protocols, reducing that naturalness present in the typical places of teaching and the educational relationship between students and teacher, an element that to date still constitutes one of the major limitations of the application of some neuroscientific techniques in educational contexts and that needs to be addressed with less invasive mobile devices. In addition, the exact positions in which the optodes are placed to create the fNIRS channels and montage are sometimes not specified in the studies considered above (e.g., first experiment by Brockington et al., 2018) and this can make the reproducibility of the study complex (Balconi and Molteni, 2016).

Furthermore, it is worth noting that these studies mainly include high-school or university students and therefore the results may be more directly relevant for this specific population group, however, we believe that they can also be taken into account for younger student populations.

Despite the instrumental constraints necessary to preserve the scientific-methodological rigor in the application of neuroscientific tools in an educational naturalistic context, it is necessary to conduct more neuroscientific studies to deepen the relationship between teacher and student and the TB perspective. With respect to what Davidesco (2020) pointed out, namely that these methods can integrate other types of measures (such as performance tests, self-reports, and think-loud interviews) and can deepen our understanding of the learning process, we add that they can be supportive also, in the analysis of the other perspective, that is of the teaching processes. The increase of research in this context would also favor the refinement of the application of wearable and wireless tools to ensure the naturalness of the interactive dyadic exchange.

### What Does the TB Suggest for Education and Teaching? The Added Value of Neuroscience

So far, there is a lack of appropriate tools to explore the TB, while a multiplicity of tools and methods has developed to explore the learning brain. The relationship of pupils and teachers in the study of teaching could become an immense source of knowledge and research on the development of the TB from the first school cycles. In this framework, the hyperscanning paradigm could be considered a potential way to overcome this gap. By allowing the simultaneous recording of the cerebral activity of the brain of the teacher-learner dyad, this paradigm will allow collecting information on student-centered and teacher-centered processing.

Moreover, the added value of neuroscience discipline is two-fold, since it consists not only in the capability of neuroscientific paradigms to provide information related to the implicit brain-and-body activity of the interactive dyad, but also in providing a range of mobile, wireless, non-invasive, and easy-to-use tools that can be exploited outside of the laboratory in real-life contexts (i.e., the classroom) with a high level of ecological validity.

The concept and research on the TB suggest that we can support teachers by valuing this approach to the study of teaching and education in general. It is not intended to outline a specific or rigid set of best practices, nor to offer a checklist that defines levels of good teaching: the underlying assumption, in fact, lies in noting that each individual teacher takes responsibility for his or her teaching. Instead, the TB framework can facilitate on the one hand the analysis of postures, neuronal markers of evidence of the teacher in the teaching activities and help to outline a path of identification, growth, and improvement (Rodriguez and Fitzpatrick, 2014), on the other hand, the results of neuroscientific evidence on the TB can allow reading how neural architectures infer behaviors and actions also directly related to teaching in real contexts.

## Conclusions

There are still few studies on this topic, which strictly anchor on the neuroscientific level of the TB: this is perhaps due to the difficulties in applying research in real-life contexts. However, research shows that some neuroscientific markers (such as brain-to-brain coupling that, for example, characterizes constructive engagement, but not information clarification; Pan et al., 2020) allow the identification of significant relationships between specific teaching methods and positive learning outcomes. This evidence must find a spillover on educational and teaching practices as well, also to modify the way of thinking about knowledge and learning processes, typical of the pedagogical perspective.

Also, two-person educational neuroscience can help to deepen the explicit and implicit levels of teaching and conceive the interactional dynamic from the teacher's view. To do so, some limitations should be overcome. Indeed, current neuroscientific studies could be divided into research that adopts very rigid protocols, reducing the naturalness present in typical teaching places and the educational relationship between students and teacher, or do not provide all the methodological details to allow replicability. Three main measures could be taken in the field of two-person educational neuroscience to address the weaknesses of current studies in this area and to increase the interpretation and application of the results. Firstly, less invasive mobile devices (wearable and wireless tools) should be adopted to ensure the naturalness of the interactive dyadic exchange between the learner and the teacher while collecting neuroscientific data. Secondly, neuroscientific studies should be designed in collaboration between neuroscientists and pedagogists to try to get as close as possible to the naturalness of the dynamics that occur in the classroom. Finally, while it is important to specify the methodological details and procedures used in these works, the evidence gathered from these studies should also be shared and translated in terms of applicability so that pedagogists and teachers can comprehend and implement it appropriately.

For educational contexts, this theme is relevant to build more functional and effective educational teaching methods and approaches, which are based on how the human brain works (both of the student and the teacher, in particular). While for psychology, the learning process is one of the basic themes of general psychology, and is relevant as a cognitive and emotional process, however, this has been studied mainly from the perspective of the student rather than of the teacher.

While using different methods and perspectives, the focus on the TB implies the possibility that knowledge from these studies can help teachers in their classroom work with students. The TB perspective also suggests that we are faced with dynamic processes of the brain on teaching, subject to continuous modifiability, and which therefore require continuous updating and in-depth analysis. It is a field of research still not much explored; with due attention to limits and potential, it can foster new instances and questions for reflection on educational research, on didactics, on the brain, and the interaction between teacher and student.

## Author Contributions

GG and LA wrote the first draft and each section of the manuscript, contributed to the manuscript final writing and revision, read, and approved the submitted version. FC and MB contributed to the manuscript final writing and revision, read, and approved the submitted version. All authors contributed to the article and approved the submitted version.

## Conflict of Interest

The authors declare that the research was conducted in the absence of any commercial or financial relationships that could be construed as a potential conflict of interest.

## Publisher's Note

All claims expressed in this article are solely those of the authors and do not necessarily represent those of their affiliated organizations, or those of the publisher, the editors and the reviewers. Any product that may be evaluated in this article, or claim that may be made by its manufacturer, is not guaranteed or endorsed by the publisher.
